# Stress-Induced Susceptibility to Sudden Cardiac Death in Mice with Altered Serotonin Homeostasis

**DOI:** 10.1371/journal.pone.0041184

**Published:** 2012-07-18

**Authors:** Luca Carnevali, Francesca Mastorci, Enrica Audero, Gallia Graiani, Stefano Rossi, Emilio Macchi, Sergio Callegari, Alessandro Bartolomucci, Eugene Nalivaiko, Federico Quaini, Cornelius Gross, Andrea Sgoifo

**Affiliations:** 1 Department of Evolutionary and Functional Biology, University of Parma, Italy; 2 Mouse Biology Unit, European Molecular Biology Laboratory (EMBL), Monterotondo, Italy; 3 Department of Internal Medicine, University of Parma, Italy; 4 Division of Cardiology, Vaio Hospital, Fidenza (Parma), Italy; 5 Department of Integrative Biology and Physiology, University of Minnesota, Minneapolis, United States of America; 6 School of Biomedical Sciences and Pharmacy, University of Newcastle, Newcastle, Australia; University of Alabama at Birmingham, United States of America

## Abstract

In humans, chronic stressors have long been linked to cardiac morbidity. Altered serotonergic neurotransmission may represent a crucial pathophysiological mechanism mediating stress-induced cardiac disturbances. Here, we evaluated the physiological role of serotonin (5-HT) 1A receptors in the autonomic regulation of cardiac function under acute and chronic stress conditions, using 5-HT_1A_ receptor knockout mice (KOs). When exposed to acute stressors, KO mice displayed a higher tachycardic stress response and a larger reduction of vagal modulation of heart rate than wild type counterparts (WTs). During a protocol of chronic psychosocial stress, 6 out of 22 (27%) KOs died from cardiac arrest. Close to death, they displayed a severe bradycardia, a lengthening of cardiac interval (P wave, PQ and QRS) duration, a notched QRS complex and a profound hypothermia. In the same period, the remaining knockouts exhibited higher values of heart rate than WTs during both light and dark phases of the diurnal rhythm. At sacrifice, KO mice showed a larger expression of cardiac muscarinic receptors (M2), whereas they did not differ for gross cardiac anatomy and the amount of myocardial fibrosis compared to WTs. This study demonstrates that chronic genetic loss of 5-HT_1A_ receptors is detrimental for cardiovascular health, by intensifying acute, stress-induced heart rate rises and increasing the susceptibility to sudden cardiac death in mice undergoing chronic stress.

## Introduction

In humans, there is broad and unequivocal evidence of causative association between chronic psycho-emotional stress and cardiovascular disorders, including myocardial ischemia and sudden cardiac death [1], [2]. Clearly, a better understanding of the neural mechanisms linking cardiac morbidity with chronic psychological stress is a fundamental step in preclinical research for the development of efficient treatments with translational relevance for clinical practice.

Recent studies provide clear and convincing evidence that central serotonergic neurotransmission plays a role in stress-elicited cardiovascular changes [3], [4]. The neurotransmitter serotonin (5-HT) has been implicated in the control of cardiovascular function during stress, and a large body of experimental evidence regarding its site of action, receptor types and underlying mechanisms has been generated (see [5] for a comprehensive review). It appears that, among at least 14 subtypes of 5-HT receptors, it is the 5-HT-1A (5-HT_1A_) subtype receptor whose involvement in cardiac control during stress is best documented. The 5-HT_1A_ receptor has been known for two decades and consequently there has been a substantial amount of research on its distribution, localization and function [5], [6]. Of particular interest for cardiac control during stress are 5-HT_1A_ autoreceptors located on serotonergic cell bodies and dendrites in the raphe nuclei of the lower brainstem [7], [8]. This medullary raphe/parapyramidal area represents the final relay for the descending pathways to the spinal sympathetic neurons that activate the heart during stress [9]. Several studies have demonstrated that sympathetic cardiomotor neurons of the medullary raphe area are sensitive to, and could be inhibited by, local administration of 5-HT_1A_ receptor agonists [10]–[12]. Consistently with this finding, exogenous activation of these receptors via systemic administration of 5-HT_1A_ agonists attenuates stress-induced tachycardic response to various psychological stressors in rats and rabbits [13]–[15].

In addition to sympathoinhibition, it has been proposed that 5-HT_1A_ agonists may also have vagomimetic effects [14]. This could be due to the activation of 5-HT_1A_ receptors located on GABA-ergic interneurons in the nucleus ambiguous [16]. Indeed, as vagal preganglionic neurons are under tonic GABA-ergic inhibitory tone, activation of inhibitory 5-HT_1A_ receptors on GABA-ergic neurons may lead to disinhibition of cardiomotor vagal neurons and consequently increase the cardiac vagal tone [16].

More recently, Nalivaiko and colleagues [17] have found that the administration of 8-OH-DPAT (5-HT_1A_ agonist) is very efficient in suppressing not only tachycardia but also cardiac arrhythmias (ventricular and supraventricular premature beats) in rats subjected to social defeat stress.

While the effect of exogenous activation of 5-HT_1A_ receptors is well-documented, our understanding of the physiological role of these receptors is still limited. The use of genetically modified mice provides new perspectives for the study of the influence of 5-HT_1A_ receptors on the physiological control of cardiac function during stress. Recently, mice lacking 5-HT_1A_ receptors (5-HT_1A_ KO mice) have been described as more anxious in several anxiety paradigms [18]–[20]. These mice have been shown to display a more anxious phenotype not only at the behavioral, but also at the autonomic level. Indeed, consistent with pharmacological studies, stressful stimuli (saline injection, foot shock and novel environment) produced larger tachycardic responses in 5-HT_1A_ KO mice than their corresponding wild-type conspecifics [21], [22].

Therefore, the purpose of the present study was to further investigate the physiological role of 5-HT_1A_ receptors in stress-induced cardiac changes, using knockout mice. Specifically, we tested whether 5-HT_1A_ KO mice displayed exaggerated cardiac autonomic responses to different stressful stimuli (from mild to prominent), such as saline injection, restraint and social defeat. Also, we investigated whether 5-HT_1A_ KO mice were less protected at the level of the heart when exposed to chronic psychosocial stress (CPS). Provided the established role of 5-HT_1A_ receptors in attenuating stress-induced cardiac reactivity, we hypothesized that KO mice might be more prone to stress-related cardiac disturbances. Indeed, during our chronic stress paradigm 6 of 22 (27%) KO mice died from cardiac arrest, whereas all wild-type mice survived the study protocol. Thus, in our study we focused on the description and characterization of the pathophysiological mechanisms that led to the death of the KO mice.

## Methods

### Ethics Statement and Animals

All experimental procedures and protocols were approved by the Veterinarian Animal Care and Use Committee of Parma University and conducted in accordance with the European Community Council Directives of 22 September 2010 (2010/63/UE).

Experiments were performed on 3-month-old male 5-HT_1A_ KO (n = 22) and wild-type (WT, n = 17) mice, originally derived from 36 heterozygous (129S6/SvEvTac;C57BL/6;CBA mixed background) breeding pairs. The founder mice for establishing our colonies were generated [23] and obtained from the Mouse Biology Unit of the European Molecular Laboratory of Monterotondo. Mice were weaned at 3–4 weeks of age, marked by ear notching and genotyped [23]. After genotyping, the 5-HT_1A_ KO and WT mice included in our experiments were housed singly in rooms with controlled temperature (22±2°C) and lighting (lights on from 7∶00 to 19∶00 h), with food and water *ad libitium*.

### General Experimental Outline

**Figure 1:**
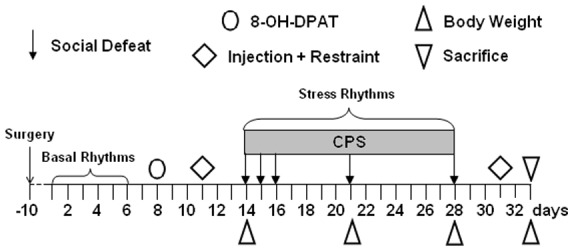
Timeline of procedures used in the current study: CPS (Chronic Psychosocial Stress).

Figure 1 shows the sequence of interventions and measurements employed in the current study; specific experimental procedures and data analysis are described in the following sections. Experiments were carried out during the inactive phase of the light/dark cycle. KO and WT mice were implanted with radiotelemetry transmitters and subjected to CPS, consisting of 15 consecutive days of constant adverse sensory contact with an aggressive male and 5 defeat episodes. During the pre-stress period, animals were injected with a 5-HT_1A_ agonist, the drug R(+)-8-hydroxy-2-(di-n-propylamino)-tetralin hydrobromide (8-OH-DPAT, 0.25 mg/Kg, i.p.) (Sigma-Aldrich, Italy). Before and after CPS, mice were submitted to a saline injection and restraint test. Diurnal rhythms of heart rate (HR), body temperature (T) and locomotor activity (LOC) were recorded around-the-clock in baseline and stress conditions. Body weights were measured before (day 14), during (day 21) and on the last day of CPS (day 28), as well as at sacrifice, at the same time of the day (9∶00). At sacrifice, the hearts were excised for structural, anatomical and immunohistochemical analysis.

### Detailed Procedures

#### Transmitter implantation

Radiotelemetric transmitters (TA10ETA-F20, Data Sciences Int., St.Paul, MN, USA) for recording ECG, T and LOC were implanted ten days prior to the commencement of the experiments. Surgery was performed under 2,2,2-tribromoethanol (Avertin, 250 mg/kg, i.p.) anesthesia and all efforts were made to minimize suffering. The transmitters were implanted using a modified version of the standard procedure [24]. Indeed, KO animals did not tolerate intra-abdominal implant and thus the body of the transmitter was placed subcutaneously in the dorsal interscapular region; two electrodes were then fixed respectively to the dorsal surface of the xyphoid process and in the anterior mediastinum close to the right atrium. Such electrode location guarantees high quality ECG recordings even during vigorous physical activity [24].

#### Chronic psychosocial stress (CPS)

In the present study we applied a modified version of the standard CPS paradigm [25], which is based on the classical resident-intruder test [26]. Six-month-old CD1 mice were individually housed with a female partner for 2 weeks and trained to aggressively defend their territory. These animals served as residents. Prior to CPS, the female partners of the resident mice were removed from the cages. Each experimental mouse was then introduced as intruder in the cage of a resident male; once there, it was vigorously attacked and finally subordinated by the resident animal (social defeat). After the first 5-min of social agonistic interaction (time counted starting from the first attack by the resident mouse), the two animals were divided by means of a perforated polystyrene-metal partition. Thus, the intruder mouse was protected from direct physical contact but it was in constant olfactory, auditory and visual contact with the resident (psychosocial challenge). The partition divided the cage into two parts of the same size and was removed for 5 min on days 15, 16, 21 and 28 (Figure 1) at an unpredictable time between 10∶00 and 13∶00 h. During direct physical interactions, the mice were separated by the partition if fighting escalated (when the dominant mouse persistently bit the opponent) to prevent injuries. After each session of defeat, the animals were closely inspected for any improper injury; at different times during the protocol, nine mice (KO n = 3, WT n = 6) were excluded from further procedures due to shallow skin wounds. At the end of CPS, the animals were returned to their home cages.

#### Saline injection and restraint test

This test was performed once before and once after the CPS period (Figure 1). The animals were first injected with saline solution (vol: 1ml/kg, s.c.) and then left undisturbed in their home cage. Fifteen min after injection, KO and WT mice were placed for 15 min in a cylindrical plastic restrainer fitted closely to the body size (inner diameter 4 cm; length 12 cm) and closed at both ends by removable partitions with holes for air circulation. After the test, the animals were returned to their home cage.

#### Biological rhythms

HR (bpm), T (°C) and LOC (counts per minute, cpm) were sampled around-the-clock for 120 s every 60 min in the two following phases: (i) Pre-stress, from day 1 to 6, with the animal in its own home cage, (ii) Stress, from day 14 to 28, between the 1^st^ and the 15^th^ day of CPS, with the animal in the resident’s cage (Figure 1). The three parameters were quantified as means of the 12-h inactive phase (light), and 12-h active phase (dark). For each mouse, the amplitude of HR, T, and LOC rhythms was calculated as the difference between mean values of the active and the previous inactive phase, respectively [27].

#### ECG data acquisition and analysis

Continuous ECG recordings were performed during: i) 8-OH-DPAT injection in baseline (30 min) and post-injection (30 min) conditions; ii) first defeat episode (day 14) in baseline (15 min, in the animal’s home cage), test (5 min, starting from the first attack by the resident mouse) and post-stress (15 min, mice separated by the partition) conditions; iii) fourth and fifth defeat episode (day 21 and 28) in baseline (15 min, in the resident’s home cage before partition removal), test (5 min, starting from the first attack by the resident mouse) and post-stress (15 min, mice again separated by the partition) conditions; iv) injection and restraint tests in baseline (30 min), post-injection (15 min), restraint (15 min) and recovery (30 min) conditions.

ECG waves were acquired (sampling frequency 2 KHz) on a personal computer (PC), via ART-Silver 1.10 data acquisition system (Data Sciences Int., St.Paul, MN, USA) and analysis was performed using a software package developed in our lab for quantification of time-domain indexes of heart rate variability. We quantified: (i) the mean R-R interval duration (RR, ms) and (ii) the root mean square of successive R-R interval differences (RMSSD, ms). RR represents an instantaneous measure of HR, the RMSSD reflects high-frequency, short-term variations of RR interval, which are predominantly due to parasympathetic influences [28]. RR and RMSSD calculations were performed after removal of arrhythmic events and recording artifacts. RR intervals (ms) were then converted to HR (bpm), according to the formula: 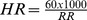.

#### ECG analysis of KO mice’s deaths

Six KO mice were found dead during the CPS period. Their ECG traces were analyzed using custom-written software during the last day before and throughout the CPS stress period, until their death. First, each ECG (consisting of 120s-time intervals that were recorded every hour) was accurately observed off-line in order to identify arrhythmic events and recording artifacts and to characterize its morphology. Electrophysiological parameters were then measured after selection of three 2s-segments for each 120s-time interval, based upon the quality of the ECG signal. We quantified the duration of: i) R-R interval; ii) PQ segment; iii) QRS complex; iv) QT interval, and v) QTc, which is the QT interval corrected for heart rate according to the standard clinical Bazett's formula: 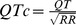.

Cardiac intervals were analyzed as follows. First, RR intervals were sorted into three categories according to their % of variation from their respective basal values: a) RR changes between 100% and 150% of the basal value (“phase 1”); b) RR changes between 150% and 200% of the basal value (“phase 2”); c) RR changes more than 200% of the basal value (“phase 3”). The other cardiac intervals were then sorted into the same three phases as their reference RR values and averaged.

#### Measurements at sacrifice

The hearts of 13 anesthetized KO and 11 WT animals were arrested in diastole by cadmium chloride solution injection (100 mM, i.v.) and excised. The two atria, the right ventricle (RV) and the left ventricle (LV) inclusive of the septum were separately weighed and fixed in 10% buffered formalin solution. LV free wall thickness and LV transversal diameters were computed for morphometric analysis using Image Pro Plus 4.0 software (Media Cybernetics, USA). The LV chamber volume was calculated according to the Dodge equation [29]. Subsequently, 5 µm-thick sections were cut, stained with Masson’s trichrome and analyzed by optical microscopy (magnification 250X) in order to evaluate the total amount of interstitial and reparative fibrosis in the LV myocardium, as previously described [30].

Auricle sections were analyzed by immunofluorescence to determine the expression of Beta-1 Adrenergic Receptor (β1) and Muscarinic 2 Acetylcholine Receptor (M2). They were incubated with the primary antibody (polyclonal rabbit anti-B1, AffinityBioreagents, Golden CO, USA, 1∶50 o.n.; monoclonal mouse anti M2, AffinityBioreagents, 1∶50 o.n.) followed by FITC or TRITC conjugated secondary antibody (Sigma, Saint Louis, Missouri, USA). Nuclei were counterstained by DAPI (4′,6-diamidine-2-phenyndole, Sigma) and cover slips mounted with Vectashield (VECTOR). Epifluorescence images examining an area of 624 to 839 mm^2^ were analyzed by microscopy (magnification 1000X). The fractional area occupied by fluorescent spots of the two different receptors and their fluorescence intensity, expressed as Integrated Optical Density (IOD), were evaluated using the Image Pro Plus 4.0 software.

### Statistical Analysis

Data are presented as means±SEM: where possible, data values have been embedded in accompanying figures. Two-way repeated-measures ANOVA, with “group” as between-subject factor was applied for results obtained from: (i) 8-OH-DPAT test (within subject factor “recording period”), ii) injection and restraint tests and social defeat episodes (within subject factor “recording period”), and iii) biological rhythms (within subject factor “time”). Follow-up analyses were conducted using Student’s “t” tests, with a Bonferroni correction for multiple comparisons. A priori Student’s “t”-tests, after controlling for homogeneity of variance via Levene test were applied for: (i) comparisons between KO and WT mice on body weight values and measures at sacrifice, and (ii) comparisons between pre-stress and chronic stress period on ECG intervals of KO mice. Death rate was compared between the two groups using Fisher’s Exact Test. P<0.05 was considered as statistically significant.

## Results

### Effects of 5-HT_1A_ Agonist Injection

**Figure 2:**
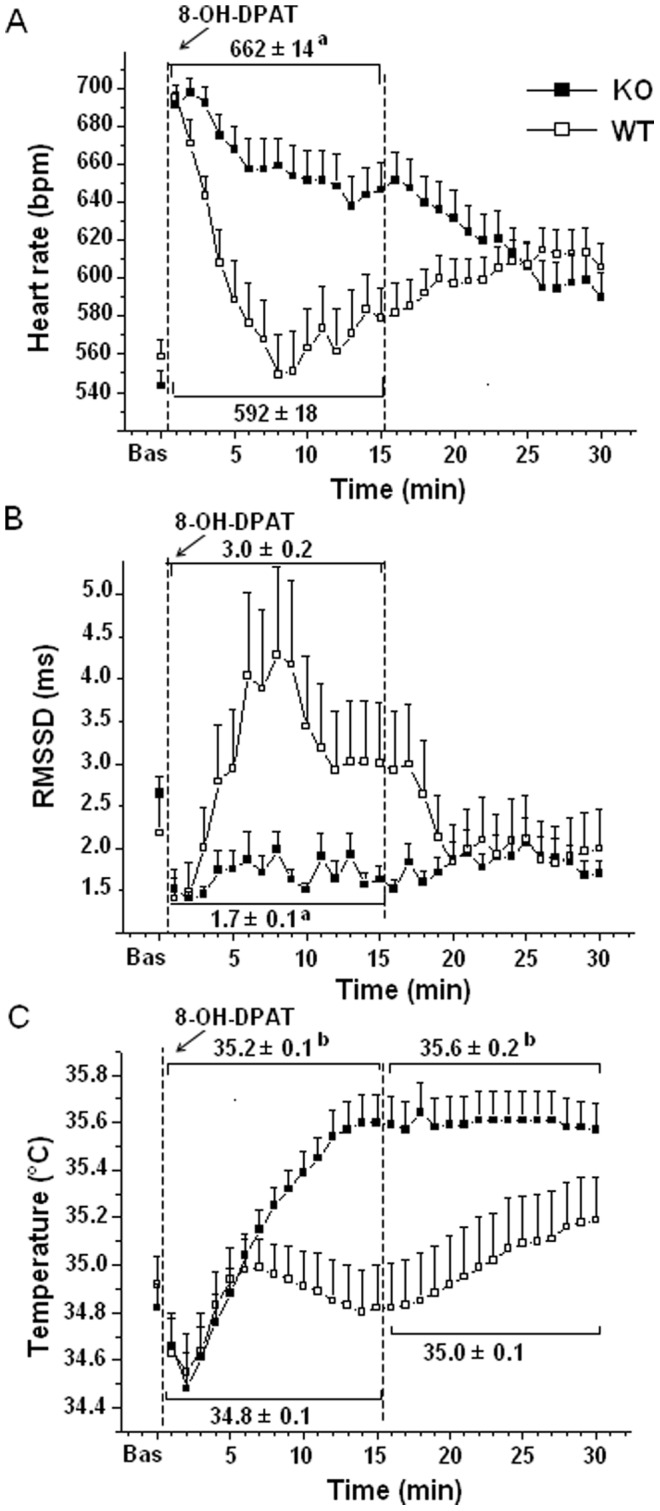
Time course of changes in HR (panel A), RMSSD (panel B), and body temperature (panel C) after 8-OH-DPAT injection in KO (n = 22) and WT (n = 17) mice. Values are means± SE. Baseline reference value is the mean of the fifteen 1-min time points in resting conditions. Results of ANOVA: significant effect of “time” (body temperature: F = 15.9, p<0.01), and “time x group interaction” (HR: F = 11.1, p<0.01). ^a^ and ^b^ = significantly different from corresponding WT value (p<0.01 and p<0.05, respectively).

Cardiac autonomic (HR and RMSSD values) and body temperature responses to 8-OH-DPAT (5-HT_1A_ agonist) administration are depicted in Figure 2. Before the injection, no differences were observed in basal values of HR, RMSSD and body temperature between the two groups (Figure 2A, B, C).

Immediately after 8-OH-DPAT injection, KO and WT mice had similar increase in HR and decrease in RMSSD values (Figure 2A, B). However, in WTs the increase in heart rate associated with drug administration reverted to the basal level within 7 min, whereas KO mice showed a much slower recovery of HR values towards basal level (Figure 2A). Similarly, WTs showed a rebound in RMSSD values within the first 4 min, whereas RMSSD values in KOs failed to return to basal level during the 30-min post injection period (Figure 2B). In addition, 8-OH-DPAT injection in KO mice provoked increase in body temperature values, whereas in WTs post-injection body temperature values did not change significantly from the basal level (Figure 2C). Mean values and statistical analysis for these parameters are presented in Figure 2.

### Cardiac Autonomic Responses to the Social Defeat Episodes

Cardiac autonomic responses (HR and RMSSD values) to the 1^st^, 4^th^ and 5^th^ defeat episode are detailed in Table 1. Differences between KOs and WTs in HR and RMSSD basal values were limited to the 4^th^ episode of defeat, where HR values of KO mice were significantly higher when compared to respective WT values (Table1). Subjecting KO and WT mice to a social defeat test provoked a similar increase in HR and a comparable reduction in RMSSD values between the two groups, which persisted during the 15 minutes that followed the cessation of the social conflict (Table1). In both groups, no differences were observed in cardiac autonomic responses to social defeat episodes across the chronic stress period. Mean values and statistical results are presented in Table 1.

**Table 1:** HR and RMSSD responses to social defeat episodes in KO and WT mice.

			Baseline(15 min)	Agonistic Interaction(5min)	Post stress 1(min 1–5)	Post stress 2(min 6–10)	Post stress 3(min 11–15)
1^st^social defeat	HR(bpm)	KO (n = 21)WT (n = 14)	556±18524±15	728±9715±9	728±14714±20	747±6727±18	757±12735±17
	RMSSD(ms)	KO (n = 21)WT (n = 14)	3.2±0.33.9±0.6	1.4±0.11.4±0.1	1.2±0.11.1±0.1	1.2±0.01.3±0.1	1.3±0.11.7±0.3
4^th^social defeat	HR(bpm)	KO (n = 16)WT (n = 14)	647±15*571±19	716±6709±13	669±18706±9	696±19720±11	723±14743±13
	RMSSD(ms)	KO (n = 16)WT (n = 14)	1.6±0.12.0±0.3	1.6±0.11.7±0.2	1.4±0.11.3±0.1	1.4±0.11.4±0.1	1.4±0.11.6±0.2
5^th^social defeat	HR(bpm)	KO (n = 15)WT (n = 12)	597±18565±16	709±9730±11	705±14740±13	730±13741±16	738±14737±20
	RMSSD(ms)	KO (n = 15)WT (n = 12)	1.7±0.22.0±0.2	1.4±0.11.4±0.1	1.5±0.11.3±0.0	1.8±0.21.5±0.1	1.7±0.21.4±0.1

Values are means ± SE.

* = significantly different from corresponding WT value, p<0.05.

### ECG and T Responses to the Saline Injection and Restraint Tests

**Figure 3:**
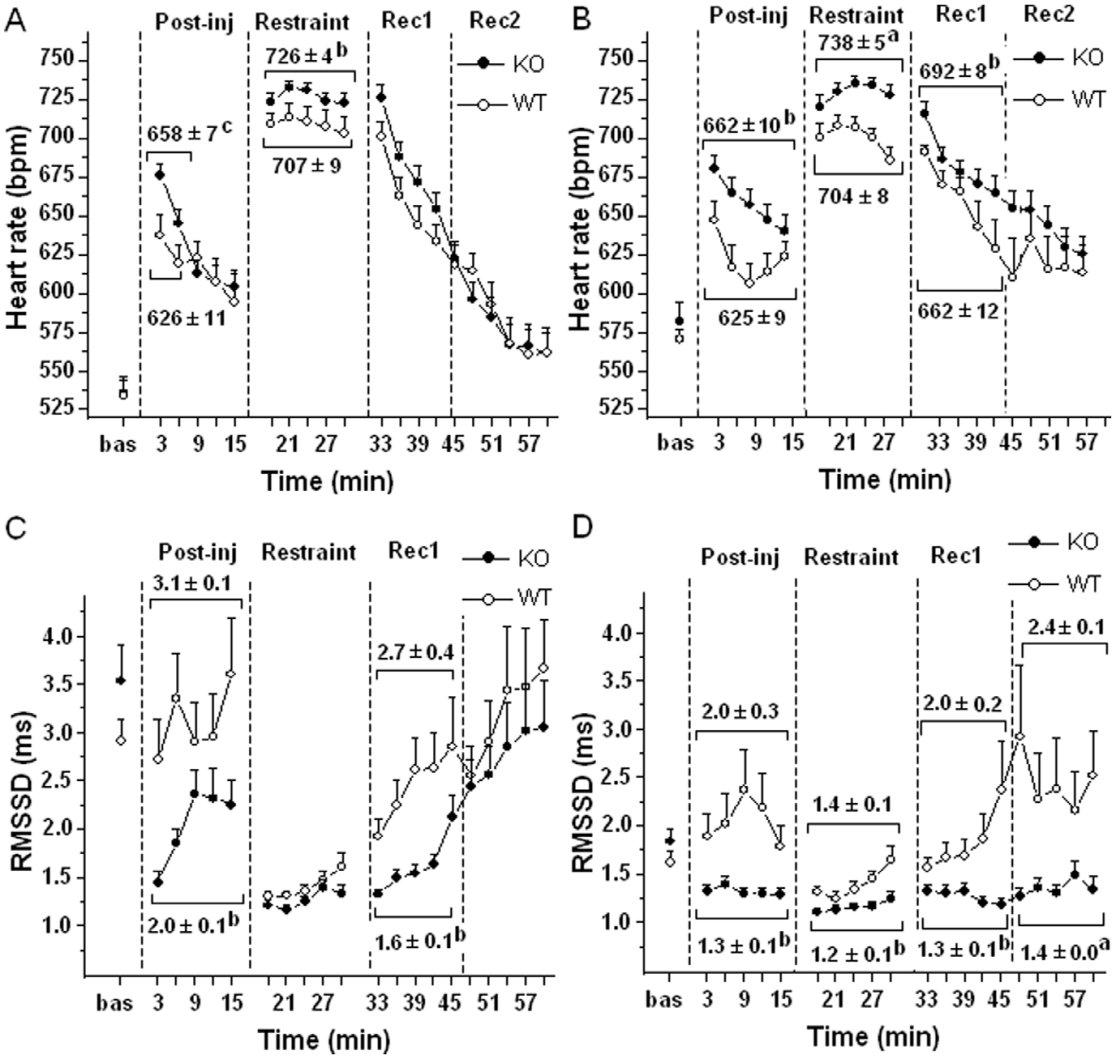
Time course of changes in HR (panel A, B) and RMSSD (panel C, D) during the saline injection and restraint tests performed before (panel A, C; KO n = 21, WT n = 16) and after (panel B, D; KO n = 14, WT n = 11) CPS. Values are means±SE. Baseline reference value is the mean of the five 3-min time points in resting conditions. In post-injection, restraint and recovery conditions each point represents the mean of 3-min time points. Statistical analysis on means of 15-min recording periods unless otherwise indicated. Results of ANOVA: significant effects of “time” (HR pre-CPS restraint: F = 17.6, p<0.01; HR post-CPS restraint: F = 16.7, p<0.01), of “group” (HR post-CPS restraint F = 5.17, p<0.05) and “time x group interaction” (RMSSD pre-CPS restraint: F = 4.03, p = 0.05; RMSSD post-CPS restraint: F = 12.01, p<0.01). ^a^ and ^b^ = significantly different from corresponding WT value (p<0.01 and p<0.05, respectively). ^c^ = Statistical analysis on the mean of the first 6min of post-injection period (KO value significantly different from corresponding WT value, p<0.05).

Once before and once after CPS, mice were submitted to a saline injection followed by a restraint stress. There were no between-group differences in basal values of HR and RMSSD before both tests (Figure 3). During the first (pre-CPS) test, the injection of saline caused cardiac acceleration in both groups, with KO mice having higher HR than WTs in the first 6 min post-injection (Figure 3A). Subjecting mice to restraint provoked potent tachycardia that was significantly larger in KOs (Figure 3A). After restraint, HR returned towards the basal levels, with no differences between groups (Figure 3A). In addition, the injection of saline provoked a fall in RMSSD values in KO mice that was not observed in WTs (Figure 3C). RMSSD values were further reduced in KOs during restraint, but the absolute values did not differ from those observed in WTs (Figure 3C). During the first 15 min of the recovery phase, RMSSD values were significantly lower in KOs than WTs, whereas no differences were observed in the following 15 min (Figure 3C).

When the test was repeated after CPS, KOs showed higher HR and lower RMSSD values than WTs after the injection of saline, during the restraint and the recovery phase (Figure 3B, D).

Mean values and statistical analysis for these parameters are presented in Figure 3.

In addition, KO and WT mice had similar basal values of T prior to the two tests (pre-CPS test: KO = 35.2±0.1°C vs. WT = 34.9±0.2°C, p = ns; post-CPS test: KO = 35.0±0.2°C vs. WT = 34.7±0.2°C, p = ns). The two groups exhibithed a comparable hyperthermia in response to the injection of saline (pre-CPS test: KO = 35.6±0.2°C vs. WT = 35.2±0.2°C, p = ns; post-CPS test: KO = 35.1±0.2°C vs. WT = 34.8±0.1°C, p = ns) and restraint (pre-CPS test: KO = 36.1±0.2°C vs. WT = 35.7±0.2°C, p = ns; post-CPS test: KO = 35.7±0.2°C vs. WT = 35.6±0.2°C, p = ns).

### Biological Rhythms

No differences between KO and WT mice were found in baseline diurnal values of HR (light: KO = 536±15 bpm vs. WT = 542±12 bpm, p = ns; dark: KO = 592±7 bpm vs. WT = 598±8 bpm, p = ns), T (light: KO = 34.7±0.1°C vs. WT = 34.6±0.1°C, p = ns; dark: KO = 35.2±0.1°C vs. WT = 35.0±0.0°C, p = ns) and LOC (light: KO = 4.3±0.4 cpm vs. WT = 4.2±0.5 cpm, p = ns; dark: KO = 8.0±0.6 cpm vs. WT = 8.1±1.0 cpm, p = ns).

**Figure 4:**
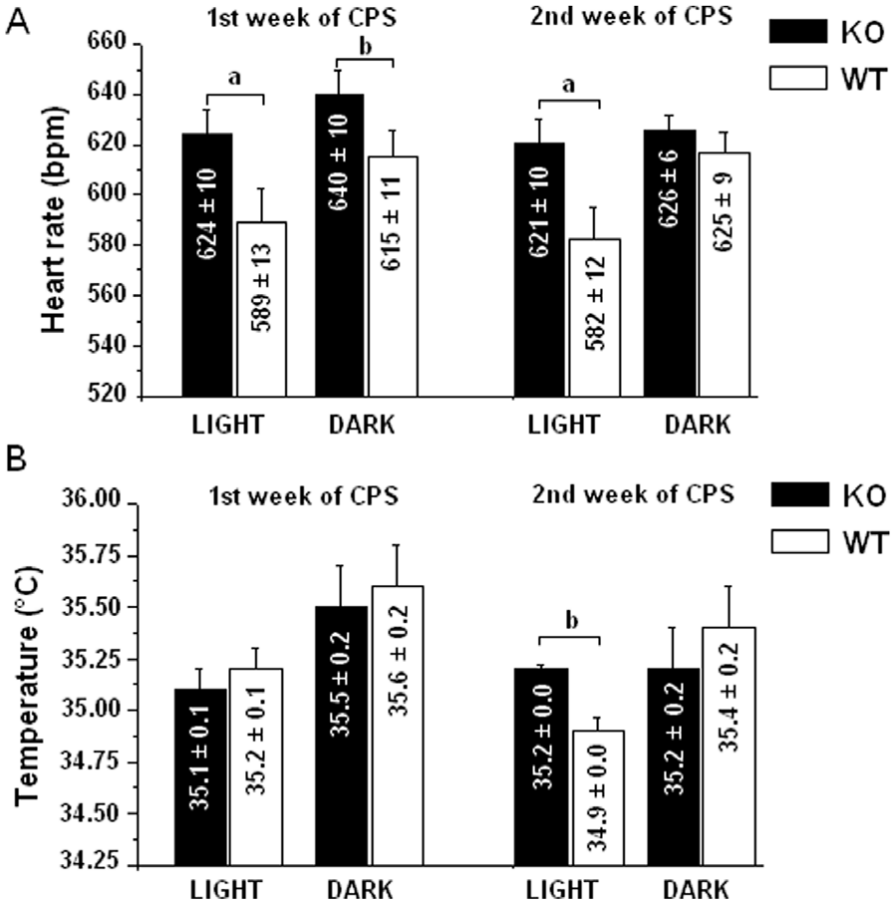
HR (A) and T (B) values in KO (n = 15) and WT (n = 12) mice during the light and dark phases of CPS. Results of ANOVA: significant effects of “group” (HR: F = 7.7, p<0.05; T: F = 34.79, p<0.01) and “group × time” interaction (HR: F = 20.76, p<0.01; T: F = 4.34 p = 0.05). ^a^ and ^b^ = significantly different from corresponding WT value (p<0.01 and p<0.05, respectively).

During the first week of CPS, KOs had significantly higher values of HR than WTs both during the active and inactive phases of the light-dark cycle (Figure 4A), whereas no group differences were observed for the values of T (Figure 4B) and LOC (light: KO = 4.6±0.6 cpm vs. WT = 4.3±0.4 cpm, p = ns; dark: KO = 6.0±0.8 cpm vs. WT = 5.6±0.7 cpm, p = ns).

During the second week of CPS, KOs had higher values of HR and T than WTs during the inactive phase of the light-dark cycle (Figure 4A, B). Consequently, KO mice had smaller HR and T rhythm amplitude than WTs (HR: KO = 7.3±5.1 cpm vs. WT = 38.1±5.6 cpm, t = −4.1, p<0.01; T: KO = 0.04±0.10°C vs. WT = 0.48±0.10°C, t = −3.0, p<0.01). In the same period, no changes were observed for LOC values between the two groups (light: KO = 3.8±0.6 cpm vs. WT = 3.2±0.6 cpm, p = ns; dark: KO = 4.9±0.6 cpm vs. WT = 5.4±0.7 cpm, p = ns).

Statistical results for HR and T parameters are presented in Figure 4.

### Body Weight

Body weight changes during CPS were calculated in animals that reached the end of the stress protocol (KO = 13; WT n = 11). KO and WT mice had similar body weight before the onset of CPS (day 14: KO = 27.9±0.5 g vs. WT = 27.3±0.5 g; p = ns). During CPS (day 21), KOs showed a reduction in body weight compared to the basal level that was not observed in WTs (KO = −0.4±0.5 g vs. WT = +2.1±0.4 g; t = −2.7, p<0.05). On the last day of CPS, the body weight of KO and WT mice did not differ (KO = 29.1±0.8 g vs. WT = 30.0±0.4 g; p = ns).

### KO Mice’s Death

**Figure 5:**
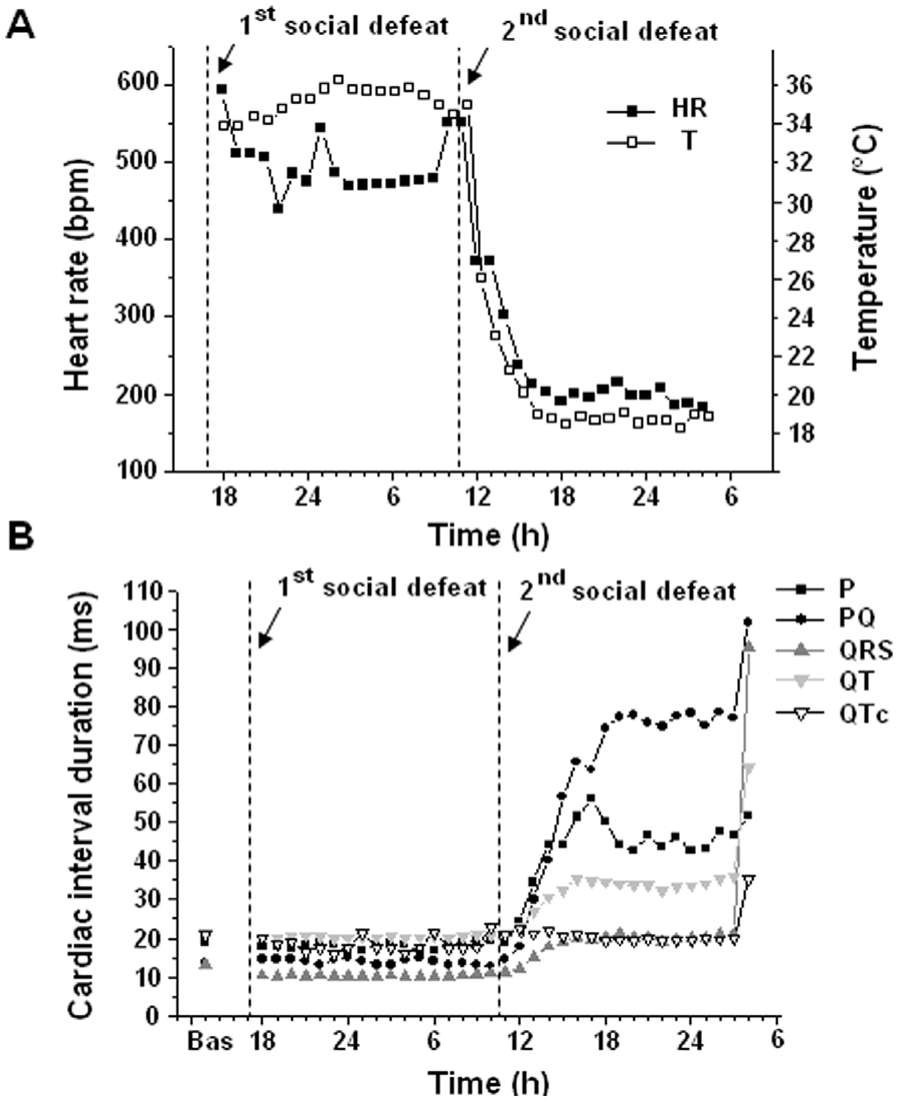
Example of HR, body temperature (panel A) and ECG intervals (panel B) changes in a representative KO mouse, from the onset of CPS to its death.

All WT mice survived the study protocol, whereas 6 of 22 (27%, p<0.05) KO mice were found dead at different times during CPS, with no evident signs of injury. Two mice died a few hours after the first social defeat, one mouse died the day after the second defeat, two mice died after the third defeat (one day and two days after, respectively), and the last mouse died a few hours after the fourth defeat episode. We observed in these mice a significant and progressive reduction in HR and T values that eventually led to cardiac arrest (Figure 5A and Table 2). Bradycardia and hypothermia occured toghether, with a similar time course (Figure 5A). Similarly, P wave, PQ segment, QRS complex and QT interval durations gradually lenghtened, whereas QT intervals corrected for heart rate (QTc) did not significantly change from the basal level (Figure 5B and Table 2).

**Table 2:** Cardiac interval changes in the six KO mice that died during CPS.

	Before CPS	CPSPhase1	CPSPhase2	CPSPhase3
RR	108.1±1.3	108.0±7.0	187.2±11.8a,b	308.0±22.2a,b,c
P	19.3±0.2	19.3±0.5	32.3±3.0a,b	45.7±4.6a,b
PQ	14.8±0.7	15.2±0.6	32.4±7.7a,b	49.6±7.3a,b
QRS	12.3±0.7	11.1±0.3	16.4±1.3a,b	25.9±4.1a,b
QT	20.8±0.4	21.5±0.7	32.3±3.5a,b	47.2±8.8a,b
QTc	20.0±0.4	21.0±1.9	23.7±2.4	27.3±5.6

Values are means ± SE, expressed in ms.

Phase 1: RR changes between 100% and 150% of the pre-CPS value; phase 2: RR changes between 150% and 200% of the pre-CPS value; phase 3: RR changes more than 200% of the pre-CPS value.

a = significantly different from corresponding ‘before CPS’ value, p<0.05.

b = significantly different from corresponding ‘phase1’ value, p<0.05.

c = significantly different from corresponding ‘phase2’ value, p<0.05.

### Cardiac Anatomy and Morphometry

#### Cardiac anatomy

As shown in Table 3, no differences were observed between KOs and WTs with respect to the weight of the heart and ventricular chambers. Also, linear LV parameters, LV chamber volume and LV mass-to-chamber volume ratio did not differ between the two groups.

**Table 3:** Gross cardiac characteristics and myocardial fibrosis in the left ventricle, in Knockout (n = 13) and Wild type (n = 11) mice.

	Knockout	Wild type
BW (g)	26.1±1.4	28.9±0.6
HW (mg)	134.9±3.8	142.6±3.1
HW/BW	0.01±0.00	0.00±0.00
LVW (mg)	106.7±2.7	113.0±3.0
LVW/HW	0.8±0.0	0.8±0.0
RVW (mg)	28.2±1.9	29.7±2.2
RVW/HW	0.2±0.0	0.2±0.0
LV Chamber Volume (mm^3^)	32.0±1.9	31.0±2.7
LV Diameter (mm)	3.0±0.1	2.9±0.1
LV Thickness (S+LV) (mm)	1.3±0.1	1.3±0.1
RV Thickness (mm)	0.5±0.0	0.5±0.0
LV Fibrosis (%)	5.1±1.3	3.5±1.0
LV Interstitial fibrosis (%)	4.8±1.3	3.2±1.1
LV Perivascular fibrosis (%)	0.3±0.1	0.3±0.1

BW: body weight; HW: heart weight; LVW: left ventricular weight; RVW: right ventricular weight.

S: septum.

Values are reported as mean ± SEM.

#### Tissue morphometry

The total amount of LV myocardial fibrosis was somewhat larger in KO mice, although no statistically significant difference was observed compared to WT mice (Table 3). Also, the volume fraction of myocytes was similar between groups (KO = 87.6±1.6% vs. WT = 89.3±1.9%, p = ns).

### Cardiac Receptor Density

The IOD as well as the fractional area occupied by cardiac auricle β1 adrenoreceptors was somewhat larger in KOs compared to WTs, although the differences did not reach significance (IOD: KO = 22.3±4.8 vs. WT = 18.7±4.2 arbitrary units, p = ns; fractional area: KO = 1.7±0.3 vs. WT = 1.4±0.4%, p = ns). On the contrary, KOs showed significantly larger distribution of cardiac M2 cholinoreceptors when compared to WTs, as expressed by the respective IOD (KO = 136.6±19.0 vs. WT = 63.6±16.0 arbitrary units, t = 2.8, p<0.05) and the fractional area occupied by these receptors (KO = 11.4±0.2 vs. WT = 5.6±0.1%, t = 3.2, p<0.05).

## Discussion

**Figure 6:**
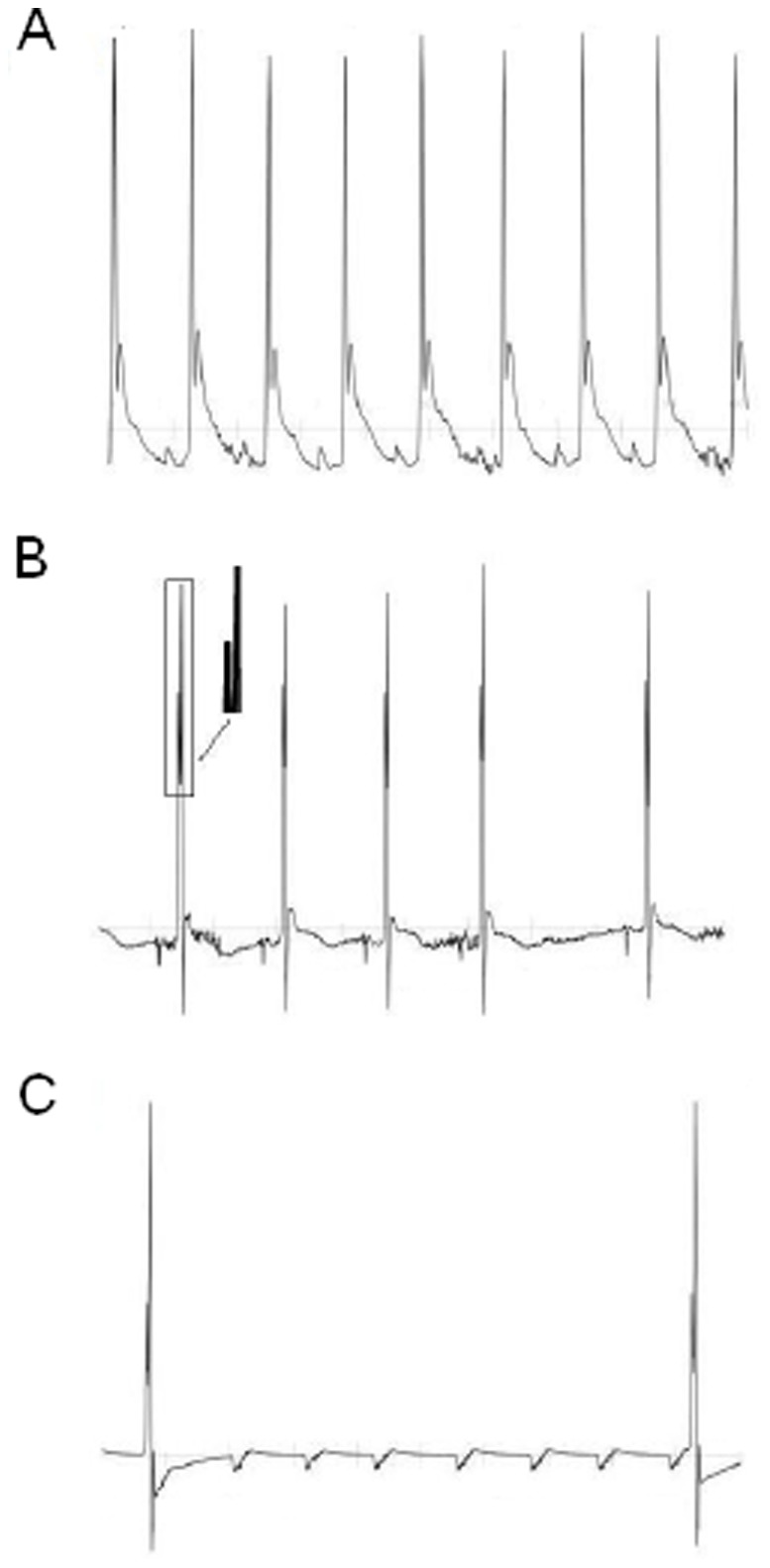
Example of ECG traces in a representative KO mouse that died during the CPS period. A: normal ECG (length of recording:1s); B: ECG with notched QRS complex (2s); C: ECG before mouse’s death with third-degree AV blocks (6s).

The present results indicate that under basal, non-stress conditions, KO mice for the serotonin 1A receptor did not differ from their WT conspecifics in any cardiac parameter. This suggests that in resting conditions either 5-HT_1A_ receptors do not play a modulating role in cardiac function or sufficient compensations were present in the KO mice. However, when the serotonergic system was challenged, certain abnormal responses became evident. Indeed, when the animals were tested in stressful conditions (saline injection and restraint), 5-HT_1A_ KO mice showed larger tachycardia and reduced vagal modulation compared to WTs. Noteworthy, 5-HT_1A_ KO mice were susceptible to sudden cardiac death when submitted to chronic psychosocial stress: specifically, six KOs developed severe bradycardia and profound hypothermia and eventually died from cardiac arrest. In these mice, cardiac interval (lengthening of P wave, PQ segment and QRS interval duration) and morphological ECG (notched QRS complex, Figure 6) changes indicated the occurrence of myocardial ischemia and severe alteration of the myocardial conduction system function.

### Effects of Exogenous Activation of 5-HT_1A_ Receptors

In WT mice, exogenous activation of 5-HT_1A_ receptors via administration of 8-OH-DPAT reduced the stress-induced tachycardia usually observed in these mice [21]. This result is in full agreement with earlier studies conducted in rats and rabbits [13], [15]. Traditional interpretation of the anti-tachycardic effect of 8-OH-DPAT is that the drug activates inhibitory 5-HT_1A_ receptors located in the presympathetic cardiomotor neurons in the medullary raphe. Our results suggest that a central sympatholytic effect is not the only mechanism responsible for this effect. Indeed, the rise in RMSSD values (vagal index) observed in WT mice after 8-OH-DPAT injection indicates that the drug increased cardiac vagal drive, presumably via disinhibition of cardiomotor vagal neurons in the nucleus ambiguous [16]. It is likely that the antitachycardic action of 8-OH-DPAT was due to both sympatholytic and vagomimetic actions.

8-OH-DPAT is also a well known hypothermic agent [31]. In WT mice, we found that the administration of the drug entirely prevented the hyperthermic response typically elicited by injection-stress in mice [21]. Previously, it has been shown that the anti-hyperthermic effect of 8-OH-DPAT is mediated by activation of inhibitory 5-HT_1A_ autoreceptors located on presympathetic neurons in the medullary raphe area [6], [32], the same brainstem region that contains presympathetic cardiomotor neurons [9]. The likely mechanism underlying this phenomenon is the inhibition of presympathetic vasomotor neurons and neurons innervating the brown adipose tissue (BAT). Consequently, stress-induced hyperthermia is reduced, due to increased heat dissipation from the tail and reduced thermogenesis from the BAT [33]. The lack of effects of 8-OH-DPAT in KOs is strong evidence of the absence of 5-HT_1A_ receptors in these mice.

### Effects of Acute Stress Exposure in 5-HT_1A_ KO Mice

Depending on the type and/or intensity of the stressor (saline injection or restraint), KO mice showed more prominent cardiac stress responses than WTs, as indicated by the larger tachycardia and fall in vagal tone (reduced RMSSD index) exhibited by KOs under stress conditions (pre-CPS test). These effects are likely a reflection of decreased serotonergic autoregulation in brain areas that control the heart during stress. In particular, the exaggerated stress-induced tachycardia observed in KOs may be due to an excessive activation of presympathetic neurons in the medullary raphe, due to deletion of the inhibitory effect of 5-HT_1A_ autoreceptors normally expressed by these cells. These results are a very important confirmation of the physiological role of 5-HT_1A_ receptors in the modulation of cardiac autonomic responses to stress indicated by previous pharmacological studies.

When the intensity of the stressor was increased by subjecting the mice to a social defeat stress, no differences were found in the magnitude of the tachycardic stress response between KO and WT mice. A possible reason why responses were similar in the two groups is likely a kind of ‘ceiling effect’ (i.e. HR reached its physiological maximum: 750 bpm in the mouse). We suppose that because tachycardia during social defeat is associated with vigorous struggling, it reaches sub-maximal or maximal levels and is thus similar in all animals, so that it no longer reflects differences in the autonomic outflow to the pacemaker region. It may be that mild stressors are more suitable for revealing differences in tachycardic responsivity, as induced by 5-HT_1A_ receptors-mediated changes in cardiac sympathetic drive.

### Effects of CPS Exposure in 5-HT_1A_ KO Mice

KOs and WTs did not show any pattern of habituation of cardiac responses to repeated social defeat episodes over the CPS period, confirming the highly aversive properties of this paradigm.

KO mice that survived the study protocol showed body weight loss during the first week of the CPS, whereas WT mice had a regular body weight growth. The weight loss in KO mice was not linked to increased locomotion. Our hypothesis is that during the first week of CPS, when most of the social defeat episodes occurred, KO mice were possibly more sensitive to the threat of being attacked by the resident mice while they were separated from them by a partition (psychosocial challenge). The body weight loss in KOs could then result from an inability to suppress a fearful behavioral response that was incompatible with feeding [34]. 5-HT_1A_ mice have been shown to be more fearful in a number of conflict tests [18]–[20], and an increased fear of aversive environment (i.e. sensory contact with an aggressive conspecific in the present study) could explain their body weight loss, confirming the important role of this receptor in modulating anxiety.

During CPS, 5-HT_1A_ KO mice displayed an enhanced anxious-like response not only at the behavioral, but also at the autonomic level. This is evidenced by the higher values of HR during both light and dark phases of the diurnal rhythm exhibited by KOs compared to WTs. Indeed, changes in emotional states, including anxiety, are highly associated with stress related increases in HR and body temperature both in humans [35], [36] and animals [6], [37].

### KO Mice’s Death During CPS

KO mice showed a susceptibility to develop stress-induced fatal abnormalities of cardiac function. Indeed, 6 of 22 mice (27%) died at different times during CPS, whereas all WT mice survived the study protocol. Death was clearly stress-related as no KO mice died before or after the end of CPS and we are not aware of any documented sudden cardiac death in colonies of 5-HT_1A_ receptor KO mice.

ECG analysis of KO mice’s death during CPS revealed that the first symptom of cardiac malfunction was a severe and progressive reduction in HR that eventually led to cardiac arrest. The bradycardia was correlated to a constant lengthening of ECG indices of conduction (P wave, PQ segment and QRS interval duration). After the first signs of bradycardia, the morphology of the ECG started to change in all the six mice, with QRS complexes displaying a notched shape (Figure 6) that progressively became more evident over time, suggesting ventricular asynchrony. Notched QRS complex may be related to changes in intramyocardial conduction time due to ischemia-induced bundle branch blocks [38]. We also found upward and downward shifts in the ST segment, morphological ECG changes typically found during infarction or ischemia [39], [40]. Taken together, the ECG characteristics of the six KO mice seem to indicate the occurrence of myocardial ischemia and the development of a severe and progressive alteration of the myocardial conduction system function. In these mice, altered conduction properties led to a large incidence of supraventricular arrhythmias, whereas ventricular arrhythmias were just occasionally noted. Also, atrioventricular blocks were frequent (Figure 6) and eventually third-degree atrioventricular blocks led to cardiac arrest and death.

In all mice bradycardia was accompanied by profound hypothermia. Whether the hypothermia was a cause or a consequence of the bradycardia is difficult to state, as the relationship between HR and temperature has never been systematically studied in mice. Prolonged asystole similar to that observed in our mice and other rhythm disturbances have been described in humans at temperatures below 32°C [41]. In our mice, near death body temperature was approximately 20°C. Berne [42] found that in dogs with body temperature of 19°C the magnitude of coronary blood flow is approximately one-fourth than normal. However, when the coronary blood flow was artificially increased from 200 to 900 per cent, no changes occurred in HR, suggesting that at low temperatures the cardiac tissue is supplied with an adequate volume of oxygen. Consequently, myocardial ischemia due to a drastic reduction in coronary blood flow may not be the prime causative factor in KO mice’s death as there is no functional cardiac abnormality directly attributable to diminished coronary flow. On the other hand, hypothermia alters myocardial conduction and often results in progressive bradycardia [43]. Thus, hypothermia-induced impairment of myocardial conduction could well be the crucial pathogenetic link in the sequence of events leading to KO mice’s death.

The alternative hypothesis is that the initial cause may have been bradycardia. When HR is slow the cardiac output is reduced, and low temperatures may be the result of compromised heat distribution from areas of thermogenesis to the rest of the body. Bradycardia is observed in patients in the presence of ongoing myocardial ischemia due to coronary spasm [44], the latter being generally believed to occur in response to an excessive release of catecholamines [45], [46]. Interestingly, the six KO mice died after a social defeat episode. It may be speculated that a larger release of catecholamines, due to the elimination of the sympatho-inhibitory effect of 5-HT_1A_ receptors, took place in KO mice during stress exposure, provoking acute coronary spasm and consequent severe myocardial hypoxia that eventually led to cardiac arrest. However, we do not have direct confirmation of catecholamine involvement in KO mice’s death. Indeed, we did not observe ventricular tachyarrhythmias that could have been expected if excessive ventricular sympathetic hyperactivity was combined with superimposed bradycardia.

As 5-HT_1A_ receptors are involved in the modulation of both cardiac and thermal responses to stress, we do not exclude the possibility that stress compromised both cardiac function and thermoregulation in KO mice.

### Long-term Effects of CPS in 5-HT_1A_ KO Mice

Three days after the last episode of defeat, mice were resubmitted to a saline injection and restraint stress test. Similarly to the pre-CPS test, the surviving KO mice showed larger tachycardia and lower vagal activity (lower RMSSD values) than WTs in response to both stress conditions, although the differences between the two groups appeared to be more consistent during this last test compared to the pre-CPS test. Also, after restraint KO mice showed slower recovery of HR to the basal level than WTs and sustained vagal withdrawal (RMSSD index) that lasted throughout the recovery period, two responses that were not observed in the pre-CPS test. It thus appears that exposure to CPS may have permanently altered the stress response system of 5-HT_1A_ receptor KO mice, sensitizing them to later stress, and leading to enhanced cardiac stress responsiveness.

At the end of the experiment, a larger down-regulation of β1 adrenoreceptors could have been expected in response to chronic excessive activation of presympathetic neurons in 5-HT_1A_ receptor KO mice compared to WT counterparts. Actually, KO mice did not differ for β1 adrenoreceptor expression in the heart auricles, but exhibited a two-fold increase in the expression of cardiac M2 cholinoreceptors compared to WTs. This up-regulation of M2 receptors observed in KO mice might represent a compensatory mechanism, occuring secondarily to an excessive shift of the autonomic balance toward sympathetic dominance and a consequent reduction in acetylcholine release from parasympathetic nerve endings.

At sacrifice, KO mice had similar cardiac structural features and total amount of myocardial fibrosis compared to WTs. A limitation of this study is the lack of control groups (no chronic psychosocial stress) for KO and WT mice that would allow evaluating the impact of CPS itself on these parameters. However, we do not rule out the possibility that the surviving KO mice may have already developed some compensatory changes over their life in response to their altered genetic makeup, thus complicating the interpretation of the data obtained at the end of the experiment.

### Significance and Perspectives

Preclinical pharmacological studies have demonstrated that exogenous activation of 5-HT_1A_ receptors decreases heart rate. In this study, we have confirmed the important role played by these receptors in the modulation of the cardiac control during stress, by demonstating that 5-HT_1A_ KO mice show more prominent cardiac autonomic responses to acute stress and adverse behavioral and physiological outcomes when exposed to a paradigm of chronic stress. Our results indicate for the first time that chronic genetic loss of 5-HT_1A_ receptors is detrimental for cardiovascular health, by increasing the risk of fatal cardiac events in mice undergoing chronic stress.

In humans, it is widely known that physical and emotional stress can lead to myocardial ischemia and sudden cardiac death. The present results, although obtained in an “extreme” animal model (knockout mice), could shed light also on human conditions of reduced density/sensitivity of these receptors that might represent an important substrate for increased susceptibility to cardiac dysregulation under chronic stress conditions.
